# Successful catheter ablation of a left posterolateral accessory bypass tract and periinterventional management in a patient with MELAS syndrome

**DOI:** 10.1007/s00399-022-00881-9

**Published:** 2022-07-08

**Authors:** Andreas Goette, Sybille Brandner, Michal Jakub Wojcik, Christian Berger, Matthias Hammwöhner

**Affiliations:** Department of Cardiology and Intensive Care Medicine, Medizinische Klinik II, St. Vincenz Hospital, Am Busdorf 2, 33098 Paderborn, Germany

**Keywords:** Atria, Mitochondria, Genetic disease, Arrhythmia, Therapy, Vorhöfe, Mitochondrien, Genetische Krankheit, Arrhythmie, Therapie

## Abstract

MELAS syndrome is defined as a combination of mitochondrial myopathy, encephalopathy, lactic acidosis and stroke-like episodes resulting from mutations in mitochondrial DNA. All medical interventions in these patients appear challenging due to a high risk of lactate acidosis or anesthesiological complications. Of note, previous reports suggest that these patients have a higher incidence of Wolff-Parkinson-White (WPW) syndrome. Here, a case of successful catheter ablation of a posteroseptal bypass tract using analgosedation in a patient with MELAS syndrome combined with WPW syndrome is presented.

## Abstract (de)

Das MELAS-Syndrom ist eine Kombination aus mitochondrialer Myopathie, Enzephalopathie, Laktatazidosen und Schlaganfallsymptomen, bedingt durch Mutationen in der Mitochondrien-DNA. Da die betroffenen Patienten gehäuft zu bedrohlichen Laktatazidosen oder anästhesiologischen Komplikationen neigen, sind alle Interventionen oder Operationen mit einem hohen Risiko behaftet. Frühere Berichte deuten bemerkenswerterweise darauf hin, dass bei Patienten mit MELAS-Syndrom die Inzidenz des Wolff-Parkinson-White(WPW)-Syndroms erhöht ist. Wir berichten in der vorliegenden Kasuistik über die erfolgreiche Katheterablation einer posterolateralen akzessorischen Leitungsbahn unter Analgosedierung bei einer Patientin mit MELAS- und WPW-Syndrom.

The combination of mitochondrial myopathy, encephalopathy, lactic acidosis and stroke-like episodes (MELAS syndrome) is a disease that results from abnormalities in mitochondrial DNA (mtDNA) [1]. The clinical presentation of MELAS syndrome may include cardiomyopathy, seizures, muscle weakness, vision and coordination impairment, dementia-like changes, hearing loss, and endocrinopathies [2]. In general, patients with MELAS syndrome carry the tRNA (Leu) A3243G mutation; however, the phenotypic manifestation varies substantially [3, 4]. The presence of ventricular pre-excitation (AVRT) and atrioventricular reentry tachycardia (AVNRT) have been reported to occur in up to 13% of in patients with MELAS syndrome. So far, there is no publication or case report in the literature reporting on successful catheter ablation of a manifest Wolff-Parkinson-White (WPW) syndrome in a MELAS patient [3]. In this context, a precise description of periinterventional procedural management including conscious analgosedation has not been reported so far.

## Medical history

The female patient was diagnosed at an age of 20 years with MELAS syndrome. Clinically, she suffered from low body height and weight (155 cm; 39 kg), pareses of the proximal musculature, elevated lactate levels at rest, hypothyreosis due to Hashimoto’s disease, oligomenorrhoea, mild deafness, mitochondrial myopathy with ragged-red-fibers on musculature biopsy, cognitive dysfunction and manifest ventricular preexcitation associated with repetitive episodes of syncope and palpitations. However, tachycardia was not recorded on a surface ECG. Genetic testing revealed a tRNA (Leu) A3243G mutation. Treatment with a beta-blocker caused substantial decline in muscular function and had to be terminated in the past.

## Observation

**Fig. 1:**
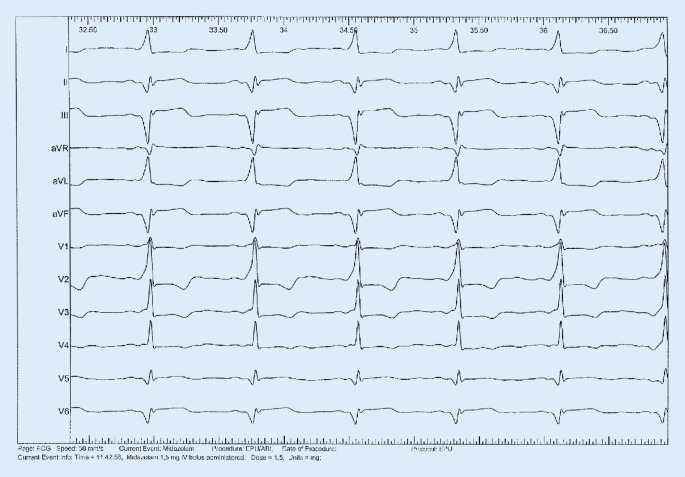
Surface ECG showing manifest ventricular preexcitation with short PQ intervals and delta waves (paper speed 50 mm/s)

Due to repetitive episodes of syncope combined with palpitations in the presence of a manifest ventricular preexcitation (Fig. 1) the indication for an invasive electrophysiological (EP) study was established.

## Diagnosis and therapy

The EP study was performed using conscious sedation. The baseline lactate level was 2.55 mmol/l. During the EP study the patient was closely monitored including oxygen saturation and systemic blood pressure. Capnometry (Medtronic, Minneapolis, MN, USA) was also performed during the procedure. Femoral veins were punctured at both sides and four femoral sheaths were inserted (3 × 6 French; 1 × 7 French). One quadripolar catheter was placed in the high right atrium (HRA), one at the His-bundle position (His), a decapolar catheter within the coronary sinus (CS) and one quadripolar catheter in the right ventricle (RVA). Electrophysiological study revealed the presence of a left lateral accessory pathway, which was mapped in transseptal technique. AV and VA conduction was documented via the pathway. Programmed atrial stimulation induced antidromic atrio-ventricular tachycardia (AVRT).

Thus, the EP diagnosis was the presence of WPW syndrome including a left posterolateral accessory pathway and inducible antidromic AVRT.

**Fig. 2:**
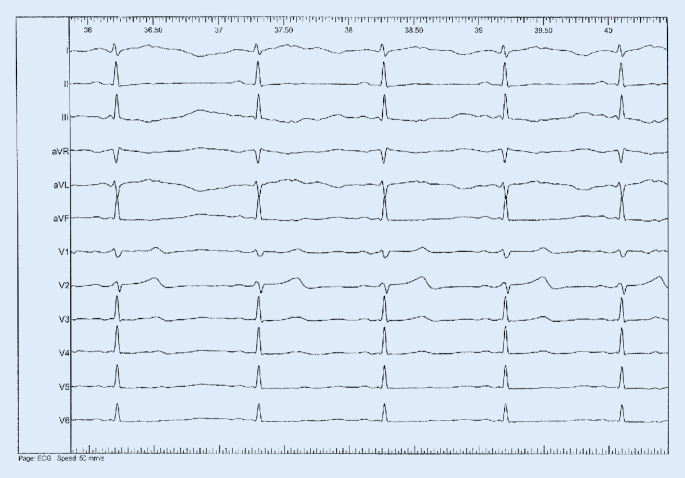
Surface ECG after ablation of the posterolateral bypass tract with normal PQ interval without a delta wave in any lead (paper speed 50 mm/s)

**Fig. 3:**
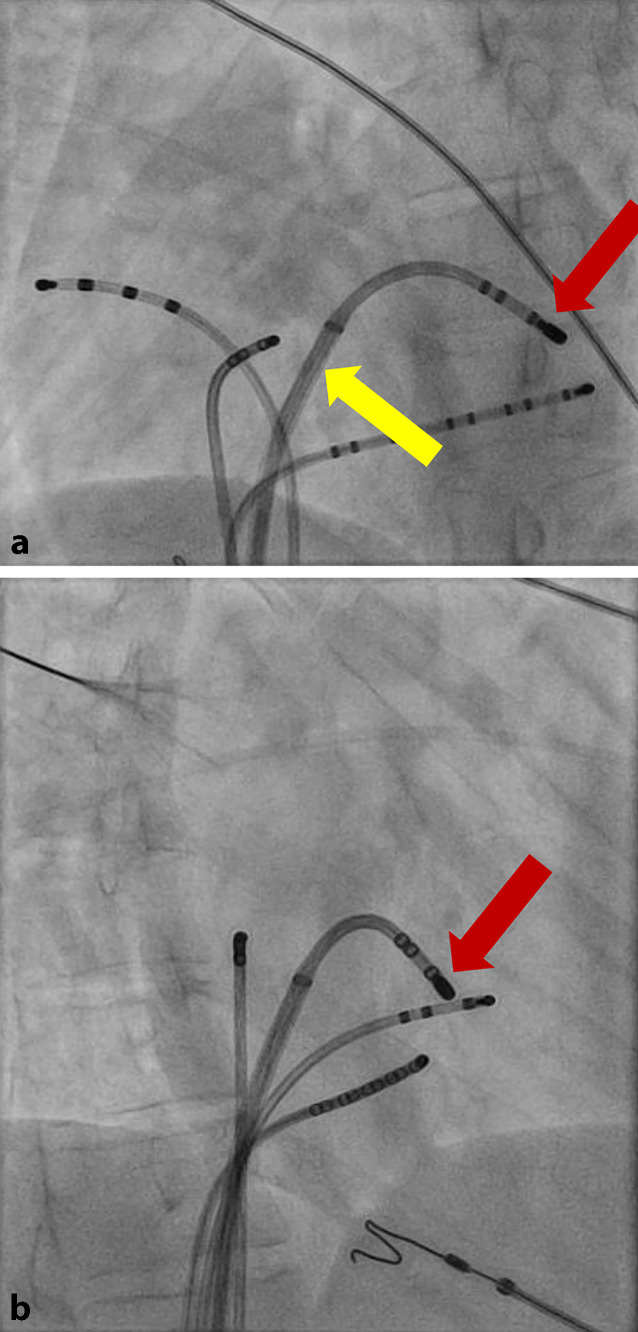
**a** Right anterior oblique (RAO 35/0) view of the successful ablation site showing the ablation catheter (*red arrow*) in the left atrium with transseptal sheath (*yellow arrow*). **b** Left anterior oblique (LAO 35/0) view of the successful ablation site showing the ablation catheter (*red arrow*)

Catheter mapping was performed using a 7-French quadripolar conventional ablation catheter. After seven applications of radiofrequency energy for each 45 s (55 Watts and 50C) conduction via the pathway was lost (Figs. 2 and 3). AVRT remained uninducible. VA conduction was lost during ventricular pacing. The normal conduction systems showed no further pathology.

During the procedure, the patient received heparin i.v. to maintain an ACT of > 300 s. In addition, the patient received midazolam as well as fentanyl i.v. with a cumulative dose of 5 × 25 µg of fentanyl (total dose of 3.2 µg per kg) and 5 × 1.5 mg of midazolam (total dose of 0.19 mg per kg). Propofol was not administered in the present case. Oxygen saturation remained above 90% during the procedure. Capnometry revealed no abnormalities in CO_2_ handling. Systolic blood pressure was about 100m Hg throughout the procedure. Lactate level (2.33 mmol/l), pH and blood glucose were checked at the end of the procedure. Electrolytes, pH and blood glucose remained within normal limits.

After the procedure, sheaths were removed within the cath lab. Pressure dressings were used at the venous puncture sites. The patient was transported to a telemetry monitoring ward and monitored until the next morning. The further clinical course of the patient was uneventful. The patient was discharged without complications, including muscular or neurological adverse effects, bleeding etc. In particular, no progress of muscular weakness was clinically detected prior to discharge.

## Discussion

The present study supports previous observations of the association between WPW syndrome and MELAS syndrome. Furthermore, the present case shows successful catheter ablation of an accessory pathway and periinterventional management including capnography-monitored deep analgosedation.

Recently, the case of a 44-year old female with a diagnosis of MELAS syndrome with the associated cardiac manifestations of left ventricular hypertrophy and atrial tachycardia was reported. That case also demonstrated cardiac involvement in MELAS syndrome. Furthermore, a larger series of 30 MELAS patients showed that 13% (4/30 patients) of the patients had signs of ventricular preexcitation on the surface ECG. Another series (Hirano et al.) noted a WPW syndrome in six of 43 patients, and in addition, cardiac conduction disturbances in three of 43 MELAS patients. In general, WPW syndrome occurs in 1.5 to 3.1 per 1000 persons in Western countries, which is substantially less than the reported incidence in MELAS syndrome. Interestingly, mutations in genes related to cellular energy metabolism, like the *PRKAG2 *gene (an adenosine monophosphate-activated protein kinase), have been linked to the development of WPW syndrome. During fetal development, altered cellular energy metabolism appears as a possible mechanism underlying the pathogenesis of abnormalities of the conduction system. Thus, MELAS syndrome including the concomitant mitochondrial pathologies may create a sort of energy depleted state, preventing normal maturation of the insulating ring, leading to persistence of abnormal conductive pathways. Nevertheless, a precise understanding of how mitochondrial diseases cause the development of WPW syndrome at molecular and cellular levels remains to be determined [1, 3, 5].

Cases of general anesthesia combined with mechanical ventilation have been described in MELAS syndrome using infusion of propofol, ramifentanyl as well as muscle relaxants. In those cases, muscle relaxant effects were antagonized with glycopyrrolate (0.4 mg) and pyridostigmine (15 mg) [1, 4–6]. In contrast to procedures in general anesthesia, the authors used conscious sedation in their case using midazolam and fentanyl. Since respiration was preserved throughout the intervention, there was no need to antagonize drug effects. In addition, pH levels remained constant without any need for an intervention. Thus, regular conscious sedation can be used in patients with MELAS syndrome. However, pH, lactate, respiration (O_2_ saturation and CO_2_ levels) should be monitored in such patients.

Thus, this is a report about successful catheter ablation of WPW syndrome including a left posterolateral accessory pathway using analgosedation in a patient with MELAS syndrome.

## Declarations

For this article no studies with human participants or animals were performed by any of the authors. All studies mentioned were in accordance with the ethical standards indicated in each case. Additional written informed consent was obtained from all individual participants or their legal representatives for whom identifying information is included in this article.
